# Prefrontal Electrical Stimulation in Non-depressed Reduces Levels of Reported Negative Affects from Daily Stressors

**DOI:** 10.3389/fpsyg.2016.00315

**Published:** 2016-03-04

**Authors:** Adelaide Austin, Gabriela M. Jiga-Boy, Sara Rea, Simon A. Newstead, Sian Roderick, Nick J. Davis, R. Marc Clement, Frédéric Boy

**Affiliations:** ^1^Department of Psychology, College of Human and Health Science, Swansea UniversitySwansea, Wales; ^2^NeuroTherapeutics Limited, Institute of Life Science, Swansea UniversitySwansea, Wales; ^3^Scientia Research Group, School of Medicine, Swansea UniversitySwansea, Wales

**Keywords:** mood, tDCS, emotion regulation, GABA antagonists, GABA agonists

## Abstract

Negative emotional responses to the daily life stresses have cumulative effects which, in turn, impose wide-ranging negative constraints on emotional well being and neurocognitive performance (Kalueff and Nutt, 2007; Nadler et al., 2010; Charles et al., 2013). Crucial cognitive functions such as memory and problem solving, as well more short term emotional responses (e.g., anticipation of- and response to- monetary rewards or losses) are influenced by mood. The negative impact of these behavioral responses is felt at the individual level, but it also imposes major economic burden on modern healthcare systems. Although much research has been undertaken to understand the underlying mechanisms of depressed mood and design efficient treatment pathways, comparatively little was done to characterize mood modulations that remain within the boundaries of a healthy mental functioning. In one placebo-controlled experiment, we applied daily prefrontal transcranial Direct Current Stimulation (tDCS) at five points in time, and found reliable improvements on self-reported mood evaluation. Using a new team of experimenters, we replicated this finding in an independent double-blinded placebo-controlled experiment and showed that stimulation over a shorter period of time (3 days) is sufficient to create detectable mood improvements. Taken together, our data show that repeated bilateral prefrontal tDCS can reduce psychological distress in non-depressed individuals.

## Introduction

One function of the dorsolateral prefrontal cortex (DLPFC) is to continuously appraise the emotional content of daily life situations, and to rapidly regulate oriented responses (Lévesque et al., 2003; Banks et al., 2007). The strong negative impact of daily stressors on current mood is well known (Bolger et al., 1989). Over time, the outcomes of this idiosyncratic evaluative and responsive process amass, and impact individuals’ emotional wellbeing and neurocognitive performance (Nadler et al., 2010; Charles et al., 2013). Here, we exploited the modulation of GABA- and glutamate-ergic neurotransmission (Stagg et al., 2009, 2011; Stagg and Nitsche, 2011; Kim et al., 2014) and cortical excitability (Romero Lauro et al., 2014) caused by transcranial Direct Current Stimulation (tDCS) to determine whether negative emotional responses to daily life stresses can be reduced in healthy individuals. tDCS involves placing two macro-electrodes on the scalp, and passing a weak regulated direct current (in the order of the mA) between them. Recent evidence from clinical research shows that repeated prefrontal tDCS in depressed patients produces measurable clinical benefits. Meta-analyses of recent open-label studies and double-blinded trials for the treatment of major depressive disorder (Fregni et al., 2006a,b; Boggio et al., 2008; Loo et al., 2010, 2012; Brunoni et al., 2011a; Dell’Osso et al., 2012), found that active prefrontal tDCS was associated, on average, with a 29.1 ± 4.6% reduction in depressive symptoms; and five of these studies detected long-lasting benefits a month after the last stimulation. In addition, Brunoni et al. (2011b) also found that 1-active tDCS was more effective than sham, 2-tDCS was as effective as Sertraline, a selective serotonin reuptake inhibitor (SSRI) antidepressant and, 3-tDCS and SSRI combined have greater efficacy than each treatment alone. This body of evidence strongly suggests that repeated daily prefrontal tDCS can be an effective tool for improving mood in depressed patient.

However, the present challenge is to understand the neurobiological underpinnings and the psychological mechanisms at play in this effect. Here, we took the original approach of studying how repeated prefrontal tDCS modulated the way non-depressed volunteers self-evaluated the emotional states consequent to life events (stressful or not). This is particularly relevant since one of the leading causal factors in depression onset is the accumulation of negative emotional states resulting from sustained or chronic exposure to stressful life events (Gandiga et al., 2006).

## Materials and Methods

### Participants

Sixty-six early adult, unmedicated, non-depressed females from Swansea University (mean age: 21.6 ± 2.3 years) participated in the experiments reported here in exchange for payment (£20) or course credit. All participants provided informed consent, were naïve to the purpose of the experiments and had no neuropsychiatric history. Participants were aware that the experimental manipulation repeatedly used tDCS neuromodulation and that they would have to complete several questionnaires, but no further specification was given as to the nature of the hypotheses. The departmental Research Ethics Committee approved all procedures. After completion of experiment 1, two participants voluntarily reported significant events that affected their current mood (passing of a relative, relationship breakup), and their data were discarded.

### Bilateral Prefrontal tDCS

**FIGURE 1:**
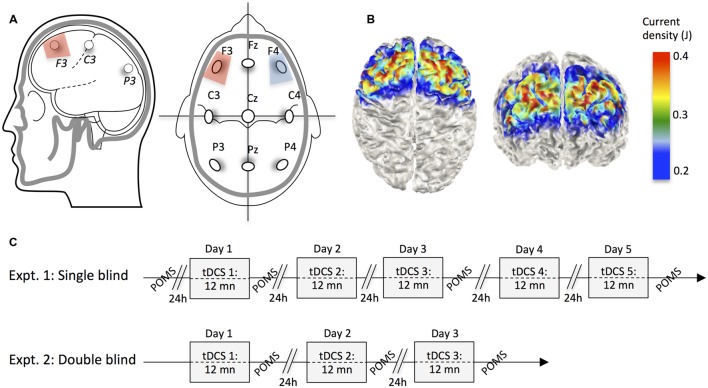
**(A)** Positioning of the stimulating scalp electrodes according to the 10–20 system nomenclature, and in reference to the main cortical fissures. **(B)** 3D numerical computation of electric fields on the surface of a cortical model for 5 × 5 cm electrodes placed on F3 and F4 head locations (Jung et al., 2013). **(C)** Timelines of experiments.

A DC stimulator (neuroConn DC stimulator, Ilmenau, Germany) delivered a 1500 mA current to the scalp via 5 × 5 cm rubber-graphite electrodes (current density: 0.06 mA/cm^2^). Impedance was automatically monitored every 5 s, and tension adjusted accordingly, so as to deliver constant current (within safety limits). In experiment 1 and 2 the anode was centered over the left F3 10–20 position (**Figure 1**). The cathode was placed over the contralateral F4 position (for similar electrodes placement see Brunoni et al., 2011b; Dell’Osso et al., 2012). Sponges soaked in 0.9% NaCl solution (Sterowash, Steroplast, Manchester, UK) were used to create a conducting medium between the scalp and electrodes. For active stimulation, the current was ramped up over 15 s and was then held at 1500 mA for 12 min, before being ramped down over 15 s. For sham stimulations, the stimulator was automatically switched off after an initial ramp-up (15 s at 0.1 mA s^-1^), plateau periods (6 s at 1.5 mA), and final ramp-down (15 s at -0.1 mA s^-1^), to create a realistic placebo control condition (Brunoni et al., 2012) that still generated the short lasting tingling sensations identical to those felt at the beginning of the active tDCS stimulations. Usually, only these very mild sensations are experienced (Gandiga et al., 2006), and when directly asked, most participants do not even perceive a difference between active and sham stimulation (Poreisz et al., 2007). Experiment 1 was single blinded (24 participants randomly allocated to either conditions in a equal proportion), whereas Experiment 2 was a double-blind randomized trial, where neither participants, nor experimenters knew whether the stimulation was active or sham (28 participants in the active condition, 14 in the sham condition).

### Mood Assessment

The Profile of Mood States (Pollock et al., 1979) questionnaire provides a rapid method of assessing transient, fluctuating active mood states. It is an instrument that is particularly well suited to the present research because of its sensitivity to change in affective states. We used the abridged scale – a 24-item questionnaire that measures mood along six dimensions: tension–anxiety, depression–dejection, anger–hostility, vigor-activity, fatigue–inertia, and confusion–bewilderment (Curran et al., 2004). Participants rated how they were currently feeling with respect to 24 words (e.g., Worn-out, Annoyed, Confused, Active, Panicky, and Unhappy) on a scale of 1 (“Not at all”) to 5 (“Extremely”). Scores at each of the factor scores, except for the vigor-activity score, was added together; and then, the vigor-activity score was then subtracted from this total to produce a general composite mood score. In Experiment 1, although participants received tDCS daily over 5 days, we limited the number of post-stimulation mood assessments by only administering the POMS every other day. A baseline measure was taken, on average, 34 min prior to the first stimulation (range 21–44 min). In Experiment 2, where participants were stimulated daily over 3 days, we administered the POMS immediately after every stimulation (**Figure 1**).

## Results

**FIGURE 2:**
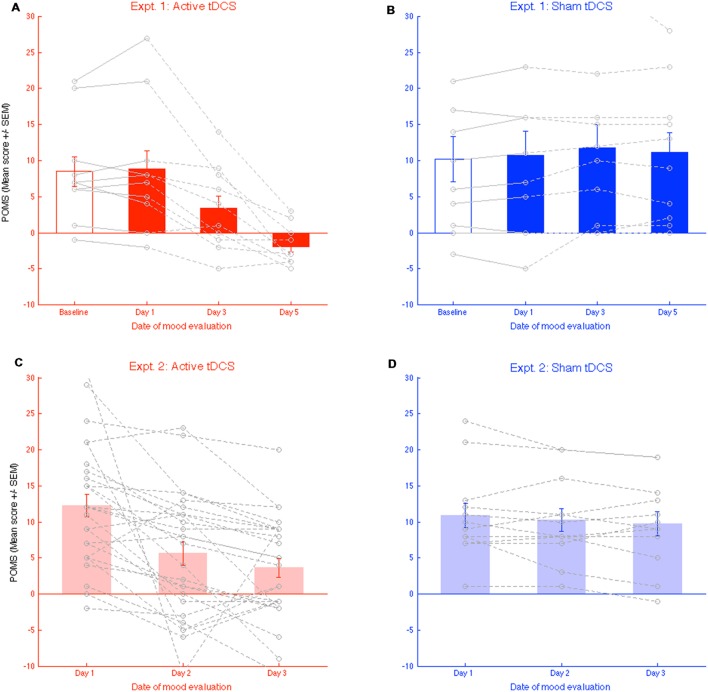
**(A,B)** Evolution of mood states self-evaluation (total score) throughout the 3 days of brain stimulation in the active, and the “sham” conditions. Grey line present individual performances in each condition. **(C,D)** Similar plots for replication experiment 2. Error bars represent the standard error of the mean (SEM).

In two experiments, we present converging evidence that series of daily bilateral prefrontal tDCS sessions positively impacted the self-assessment of mood states. In Experiment 1, we first established that, when five 12 mn daily tDCS sessions were administered, scores on the Profile of Mood States scale were improved in the active [*F*(2,22) = 20.18, *p* < 1.1e-05, $\eta_{p}^{2}$ = 0.65; **Figure 1A**], but not in the sham condition [*F*(2,22) = 1.03, *p* < 0.37; **Figure 1B**]. In the active condition, significant improvements were found between evaluations carried out each other day (all *ps* < 0.01), whereas no change was noted between sham sessions (all *p* = NS). This striking dichotomy was independently replicated in Experiment. 2, where tDCS sessions were administered on three consecutive days [active: *F*(2,54) = 17.31, *p* < 1.56e-06, $\eta_{p}^{2}$ = 0.39; **Figure 2C**; sham: *F*(2,26) = 1.59, *p* < 0.22, **Figure 2D**]. In substantive terms, the reduction in negative mood states in the two active tDCS conditions accounted for 64.7 and 39.1% of the total variations in scores in Experiment 1 and 2, respectively.

The absence of significant mood changes in the sham condition, where participants received series of 36 s 1.5 mA daily stimulations, insured that the observed negative mood reduction was not due to a learning or habituation effect, with participants (consciously or unconsciously) gradually providing less negative ratings during the mood evaluation.

**FIGURE 3:**
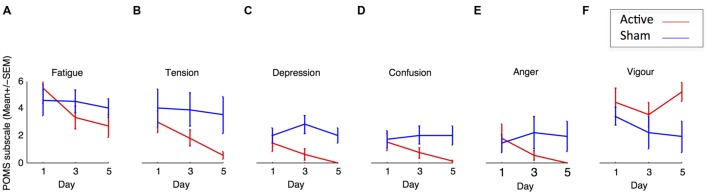
**(A–F)** Evolution of self-evaluation for each dimensions of the POMS throughout the 3 days of brain stimulation in the active and sham conditions in experiment 1. Error bars represent the SEM.

The general tendency toward mood improvement during active tDCS evidenced in the reduction in general composite mood score is logically resulting from improvements in each of the subscales. Although the design of the present research is not adapted to such subsampling of the data, we decided to still present how scores at each of the six subscales in the POMS were modulated by tDCS, without presenting any result of statistical testing (**Figure 3**). Although the argument is only descriptive, and variability is high, we note that, for all subscales except “vigor,” there is an amelioration tendency (a decrease in scores) in the active tDCS but not in the Sham condition. Interestingly, in Experiment 1, we failed to find significant changes in the mood evaluations made before (labeled “Baseline” in **Figure 2**) and after the first stimulation [Day 1 active: *t*(11) = 0.47, *p* < 0.64, sham: *t*(11) = 1.28, *p* < 0.23]. Our current research program explores these aspects, in an adapted research protocol with sufficient statistical power.

## Discussion

In two sham-controlled experiments, we found that repeated daily prefrontal tDCS sessions over 5 several days could effectively modulate how non-depressed individuals self-assess their mood states. Results show that participants experienced less psychological distress from daily stressors, a well established cause in the establishment of a negative emotional state (Bolger et al., 1989). We replicated this finding in an independent, randomized, double-blind experiment applying similar protocol and stimulation on 3 consecutive days.

To our knowledge, the present research is the first to show that the amount negative mood states, in unmedicated non-depressed individuals, can be reduced with repeated prefrontal tDCS. This is consistent with prior body of clinical research demonstrating that repeated tDCS significantly reduces symptoms of major depressive episodes (see Kalu et al., 2012 and Meron et al., 2015 for recent systematic reviews). It is also true that a few conflicting studies failed to find a reduction in psychological distress following prefrontal tDCS. However, the body of research in question either examined individuals with treatment-resistant depression, or else participant samples that were concurrently taking various medication treatments known to interact with tDCS (Loo et al., 2010; Blumberger et al., 2012; Bennabi et al., 2015). For example, the administration of GABA-agonist benzodiazepines (Lorazepam) delays, enhances, and prolonges the elevation in cortical excitability resulting from anodal tDCS (Nitsche et al., 2004), while serotonin selective reuptake inhibitors such as citalopram concurrently increases anodal effects, and transforms cathodal inhibition into facilitation (Nitsche et al., 2009; Brunoni et al., 2013; Bennabi et al., 2015).

Only a few other studies have examined the possibility of modulating mood using tDCS in healthy individuals, these however, failed to show significant effect (Koenigs et al., 2009; Plazier et al., 2012; Morgan et al., 2014). While it is difficult to discuss the absence of significant effect, we believe these could be accounted for by radically different research designs, stimulation program, electrode montages, or in the way current mood was evaluated. For example, in both experiments reported here, participants underwent either 5 or 3 consecutive days of active (or sham) bifrontal tDCS, and our conclusions are therefore founded upon comparisons between an active group and a sham group, and self reported modulation of mood occurring across days. In contrast, Plazier et al.’s (2012) goals were rather different and the research was looking for alterations in mood, following a single session of tDCS, utilizing six forms of bifrontal and bioccipital stimulation, upon the same participant. Although such an attempt is both interesting and commendable, it is difficult to conceive that biochemical alterations within the cortex or detectable effects on mood resulting from a single 20 min tDCS session would be of similar origins to mood modulation observed across days of repeated stimulation. Of importance, we also think that Plazier et al.’s (2012) way of administering mood questionnaires directly before and after the stimulation, is far from optimal: The short time period between repeated assessments, and a participants’ initial responses will likely have influenced, to some degree, latter responses to the questionnaires re-administered directly following stimulation. This observation regarding the limits of repeated questionnaire administration also applies to Koenigs et al. (2009) and Morgan et al. (2014) for the Positive Affect Negative Affect Schedule (PANAS), and the POMS, respectively. Of importance, Koenigs et al. (2009) rather placed the anode on the frontal poles bilaterally (F_p1_ and F_p2_), and used an extracephalic reference electrode, but detected no mood improvement after any of three usual tDCS conditions.

Discussion of certain methodological questions is needed to further inform future investigations. For instance, our data may have implications for the interpretation of numerous findings in which prefrontal tDCS induces cognitive improvements (e.g., Kadosh et al., 2010; Jacobson et al., 2011). Cognitive processing is affected by mood, with positive mood being associated with improved cognitive performance (Nadler et al., 2010), and since we show that tDCS reduces self reported psychological distress, it is possible that tDCS-induced cognitive improvement are actually mediated by a mood improvement (or vice-versa). Current neuromodulatory work in our team address this issue, and aims at disentangling the complex interaction between mood and cognitive performance. Another pertinent issue relates to the duration of the tDCS-induced mood modulation. In clinical studies, researchers have reported mood improvement effects to be maintained for at least one month after the last stimulation (Kalu et al., 2012). However, these studies involve a greater number of stimulation sessions (*N* = 10), over a longer period of time (2 weeks), possibly suggesting that the optimal program of stimulation needed to warrant a potentiated reduction of psychological distress in a non-clinical population still has to be determined.

Both past studies in depression, and the present work suggest that tDCS is effective in reducing depressive symptoms and psychological distress, respectively. An important question to consider concerns the identification of the neurophysiological mechanisms that are able to induce these changes. One possible explanation follows from two programs of research. One that examined the relationship between GABA levels and depression, and evidenced that GABA-agonist drugs and agents all tend to ameliorate the depressive symptomatology in human, and in animals (Kalueff and Nutt, 2007). Another, more recent body of research, showed that tDCS could lower cortical GABA and glutamate level locally (Stagg et al., 2009). Although the latter effect was obtained in regions of the frontal cortex that are not directly causally related to mood regulation, unlike the DLPFC, we believe that it provides a general framework for the generation of testable hypotheses. The details of the interaction between GABAergic neuromodulation within the DLPFC and mood regulation are likely to be complex, as the DLPFC forms part of a network involving loops through striatum and thalamus as well as numerous connections to other cortical and subcortical areas relevant for regulating mood. Similarly, the apparent contradiction between the effect of a technique which lowers levels of GABA and a general GABA deficit theory in depression has to be understood in this context, and treated with great care. It is indeed possible that the tDCS-induced local reduction in GABA concentration results in potentiated GABAergic neurotransmission along these extended networks (for similar reasoning, see discussion in Boy et al., 2011).

## Author Contributions

SR, AA, FB collected data; SR, FB analyzed data; FB, SR, ND, SN, RMC, GJB wrote or commented on different versions of the manuscript.

## Conflict of Interest Statement

The authors declare that the research was conducted in the absence of any commercial or financial relationships that could be construed as a potential conflict of interest.

FB filed the patent application no. 1503004.2 (Intellectual Property Office, Newport, UK) for a novel tDCS device. GJB, SR and FB hold shares in NeuroTherapeutics Limited, a UK registered Company. RMC is an indirect shareholder. The other authors declare that the research was conducted in the absence of any commercial or financial relationships that could be construed as a potential conflict of interest. At the time of data collection, students who gave tDCS stimulation and collected behavioral responses were blind to the hypotheses and previous findings.
